# Multicenter Retrospective Andalusian Study of the Use of Sonidegib for the Treatment of Local Advanced Basal Cell Carcinoma in Real Clinical Practice

**DOI:** 10.3390/jcm12175631

**Published:** 2023-08-29

**Authors:** Ricardo Ruiz-Villaverde, Enrique Herrera-Acosta, Andres Ruiz de Casas, Isabel M. Villegas-Romero, Fátima G. Moreno-Suárez, Francisco Vílchez-Márquez, Manuel Galán-Gutiérrez, Maria Carmen Vázquez-Bayo, Sandra Cases-Mérida, Francisco M. Almazán-Fernández

**Affiliations:** 1 Hospital Universitario San Cecilio, Instituto Biosanitario de Granada, Ibs, 18016 Granada, Spain; franciscom.almazan.sspa@juntadeandalucia.es; 2 Hospital Universitario Virgen de la Victoria Málaga, 29010 Málaga, Spain; enrique.herrera.acosta.sspa@juntadeandalucia.es; 3 Hospital Universitario Virgen Macarena, 41009 Sevilla, Spain; andres.ruiz.casas.sspa@juntadeandalucia.es; 4 Hospital Universitario Puerta del Mar, 11009 Cádiz, Spain; isabelm.villegas.sspa@juntadeandalucia.es; 5 Hospital Universitario de Jaen, 23007 Jaen, Spain; fatimag.moreno.sspa@juntadeandalucia.es; 6 Hospital Universitario Virgen de las Nieves, 18014 Granada, Spain; francisco.vilchez.marquez.sspa@juntadeandalucia.es; 7 Hospital Universitario Reina Sofía, IMIBIC, 14004 Córdoba, Spain; manuel.galan.sspa@juntadeandalucia.es; 8 Hospital Universitario Juan Ramon Jimenez, 21005 Huelva, Spain; mcarmen.vazquez.sspa@juntadeandalucia.es; 9 Hospital Universitario de Jerez, 11407 Cádiz, Spain; sandra.cases.sspa@juntadeandalucia.es

**Keywords:** sonidegib, treatment, local advance basal cell carcinoma

## Abstract

Introduction: Locally advanced basal cell carcinoma (LA-BCC) is defined as that BCC in which there is radiological confirmation of invasion of certain neighboring structures in depth and also, usually, a BCC that is of a sufficient size and invasion (although there is no radiological demonstration of deep invasion) in which surgery and radiotherapy are not adequate, are insufficient or are contraindicated to achieve the cure of the tumor, either due to characteristics of the tumor itself or of the patient. Sonidegib is indicated for the treatment of adult patients with locally advanced basal cell carcinoma that is not amenable to curative surgery or radiotherapy. Material and methods: This is a retrospective, multicenter and descriptive study in nine centers in Andalusia, Spain. Patients treated with sonidegib for >3 months for locally advanced BCC were included from 1 January 2021 to 1 January 2023. Epidemiological, efficacy and safety data were collected. Results: In the present study, a total of 38 patients were included, with a median age of 76.23 years (range 40–101). Prior treatment was surgery (31.57%; *n* = 25), radiotherapy (15.78%; *n* = 6), vismodegib (31.57%; *n* = 12). Eleven patients had not received prior treatment. LA-BCC were located in the cephalic pole, face or scalp. There was a total response in 9/38 patients (23.7%), partial response in 25/38 patients (65.8%) and no response in 4 patients (10.52%). In 6/34 patients, the dose was reduced to 200 mg every other day until it was discontinued due to adverse effects. The main adverse effects reported were dysgeusia (*n* = 8), asthenia (*n* = 8), = 6), muscle spasms (*n* = 6), alopecia (*n* = 4) and gastrointestinal intolerance (*n* = 4). Discussion: Sonidegib is the second iHh authorized for the treatment of adult patients with locally advanced BCC who are not amenable to curative surgery or radiotherapy, based on the results of the phase II clinical trial, BOLT. Sonidegib shows good effectiveness and an acceptable safety profile in routine clinical practice in the sample presented.

## 1. Introduction

Non-melanoma skin cancer (NMSC) mainly includes basal cell carcinoma (BCC) and squamous cell carcinoma (epidermoid or squamous cell) and a heterogeneous group of tumors with a lower prevalence, including Merkel carcinoma, cutaneous lymphoma and adnexal tumors [1]. The BCC represents approximately 80% of NMSC [2] and presents considerable variability in its morphology. Basal epithelial tumor cells form clusters surrounded by stroma with different growth patterns, giving rise to slow-growing BCC with the nodular and superficial subtypes, and aggressive and infiltrative growth with the morpheiform, infiltrating, micronodular and basosquamous subtypes. A single histological pattern may be present in a tumor, or a combination, which is then called mixed histology [3,4]. Solid or nodular basal cell carcinoma is the most frequent variant.

BCC is the most common skin tumor with an estimated annual incidence of 0.1 to 0.5% [1]. In Spain, the incidence of basal cell carcinoma is estimated at 113.05 (95% CI: 89.03–137.08)/100,000 person-years, counting a single tumor per person and histological diagnosis; at 253.23 (95% CI: 273.01–269.45)/100,000 person-years, counting tumors instead of people; and for the incidence of squamous cell carcinoma, 38.16 (95% CI: 31.72–39.97)/100,000 person-years [1]. It is more common in people between the ages of 60 and 70 and in men. Photoexposed areas are the most frequent locations in up to 80% of cases.

BCC is considered a locally aggressive, slow-growing disease. The presence of metastasis is rare and ranges from 0.0028% to 0.5% [5]. The onset interval is 9 to 11 years, and once metastases have occurred, 5-year survival is 10% [6].

The etiology of the BCC is multifactorial, with constitutional (intrinsic) and environmental (extrinsic) factors. Accumulated ultraviolet radiation plays a fundamental etiopathogenic role. Ionizing radiation and chemical agents (polycyclic aromatic hydrocarbons, chlorophenols and arsenic) are also considered extrinsic factors. The intrinsic factors involve genetic syndromes associated with sensitivity to ultraviolet radiation as well as xeroderma pigmentosum, Bazex-Dupré-Christol syndrome, albinism and Gorlin syndrome.

Locally advanced basal cell carcinoma (LA-BCC) is defined in a general way, as that BCC in which there is radiological confirmation of invasion of certain neighboring structures in depth and also, probably, a BCC of a sufficient size and invasion (although there is no radiological demonstration of the invasion in depth) in which surgery and radiotherapy are not adequate, are insufficient or are contraindicated to achieve tumor cure, either due to characteristics of the tumor itself or of the patient [7].

The elective treatment for any clinical form is surgical resection (Mohs micrographic surgery or conventional surgery), as it allows histological control of the tumor margins and has lower recurrence rates. The use of surgical excision for the treatment of BCC obtains cure rates close to 90% at 5 years [8]. However, if the tumor is large and located in photo-exposed areas, it tends to be treated with less invasive techniques such as photodynamic therapy, immunomodulators, topical or systemic chemotherapy, topical imiquimod, topical 5-fluorouracil, interferon intralesional therapy, cryosurgery or electrocoagulation, radiotherapy or drugs that inhibit the Hh pathway (vismodegib and sonidegib). The choice of treatment should consider the type and location of the tumor and the characteristics of the patient and risk factors for recurrence.

There is no standard treatment for patients with LA-CBC, and the therapeutic alternatives available are limited [9].

The Hh signaling pathway was observed for the first time in 1980, in the larva of Drosophila melanogaster, in which it was seen that mutations in the patched and hedgehog genes, which encode this signaling pathway, produced alterations in embryonic development. In different species, this pathway plays a fundamental role in organogenesis during embryogenesis, but in adulthood, the Hh pathway is practically inactivated, only remaining active in the hair follicle, skin and stem cells, maintaining tissue homeostasis and repair. In the Hh pathway, the Patched 1 (PTCH1) transmembrane receptor, located in the primary cilia of the cells, in the absence of ligand, inhibits the Smoothened (SMO) protein, a transmembrane receptor that acts as a signal transducer. When an Hh ligand binds to PTCH1, the repression of SMO disappears, which acts on the suppressor of fused homolog (SUFU) mediator protein, promoting the activation of glioma-associated oncogene transcription factors (GLI) and inducing the transcription of genes involved in increased survival cell and mitosis. Aberrant activation of the Hh signaling pathway is the trigger for the pathogenesis of BCC, in more than 90% of cases, but also in other types of tumors such as neuroblastomas, gliomas and rhabdomyosarcomas. Inhibition of the Hh signaling pathway is a key therapeutic target in the treatment of CBCla, for which surgery or radiotherapy are not feasible. Vismodegib and sonidegib inhibit the Hh pathway by binding to and inactivating the SMO protein, thereby suppressing tumor growth.

Sonidegib is indicated for the treatment of adult patients with locally advanced basal cell carcinoma that is not amenable to curative surgery or radiotherapy [10]. It is available as 200 mg hard capsules. The recommended dose is 200 mg sonidegib administered orally once daily (QD) at least two hours after a meal or at least one hour before the next meal, at the same time each day. If dose reduction is required, a dose of 200 mg every other day will be administered.

To sum up from the physiopathological point of view, Sonidegib is an orally bioavailable inhibitor of the Hh signaling pathway. It binds to Smoothened (SMO), a molecule of the class of G protein-coupled receptors that positively regulates the Hh pathway and eventually activates and releases glioma-associated oncogene transcription factors (GLI), which induce transcription of the Hh target genes involved in proliferation, differentiation and survival. Sonidegib that binds to SMO will inhibit Hh signaling and consequently block signal transduction [10]. It is primarily metabolized via CYP3A4, and co-administration of strong CYP3A4 inhibitors or inducers may significantly increase or decrease sonidegib concentrations.

## 2. Material and Methods

This is a retrospective, multicenter and descriptive study in 9 centers in Andalusia, Spain.

Patients that were treated with Sonidegib (Odomzo^®^, Sun Pharmaceutical Industries Europe B.V, Hoofddorp, Países Bajos) for >3 months for locally advanced BCC (European Association of Dermato-Oncology [EADO] classification stage III) were included from 1 January 2021 to 1 January 2023.

The study was approved by the Ethics Committee of Hospital Universitario San Cecilio, Granada, Spain (approval code: HUSC_DERM_008/2023).

Epidemiological, efficacy and safety data were collected. The clinical response has been evaluated following the parameters of the recently published Spanish series [10] with complete response (CR) when there was no visible tumor; partial response (PR) when the tumor decreased by at least 50% in size; stable disease (SD) when tumor reduction was less than 50%, or less than 20% increase in tumor area; progressive disease (PE), when the tumor increased in size by at least 20%.

## 3. Results

A total of 38 patients were included in the present study, with a median age of 76.23 years (range 40–101). Table 1 shows the epidemiological and clinical characteristics of the patients. In our series, there were 16 men (42.10%) and 22 women (57.90%). All the patients in our sample were Caucasian. The median weight was 76.7 kg. Moreover, 26.10% of the patients were not independent for the basic activities of daily life. Only 2 patients (5.2%) had a diagnosis of Gorlin syndrome. The total number of BCCs treated was 38 (1 for each patient, including those diagnosed with Gorlin’s Syndrome).

Previous treatment was surgery (31.57%; *n* = 25;), radiotherapy (15.78%; *n* = 6; total dose: 54–60 Gy), vismodegib (31.57%; *n* = 12). Eleven patients had not received previous treatment or other treatments (28,94%). Of the patients who underwent prior surgery, 26.31% received Mohs surgery, and the median number of surgeries performed for CBCla was three (range 1–12). In relation to the previous treatment with vismodegib, the drug was changed to sonidegib due to lack of response in all cases. The median duration of Vismodegib treatment was 6.7 months (range: 3–14 months). The washout period before starting treatment with Sonidegib was not collected by all the authors participating in the study since it depended on the internal processes of each hospital for the approval of the second inhibitor of the hedgehog pathway in all cases in which it could be verified in more than 4 weeks. Vismodegib was discontinued due to lack of partial/total response in 74% of cases at the discretion of the prescribing physician and due to side effects not tolerated by the patient in 26% of cases.

In addition, 92.10% of the patients presented an aggressive histotype (*n* = 35). All LA-BCCs were located on the cephalic pole, face, or scalp.

**Figure 1:**
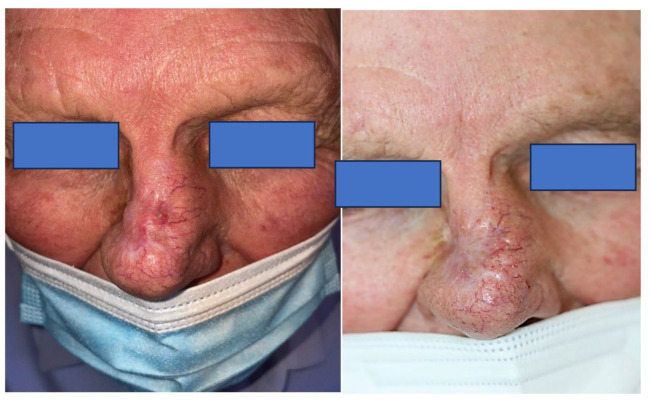
Patient number 15. (**Left**): Locally advanced basal cell carcinoma in the nose. (**Right**): Clinical evolution at 12 weeks (Partial response). The patient consented to the use of images for academic and scientific purposes. Courtesy of Dr. Fátima G. Moreno-Suarez.

Sonidegib was started as the main treatment in all cases and was discontinued at the investigator’s discretion if there was a complete response, no response or intolerable adverse effects after the tapering regimen. The initial treatment dosage was 200 mg daily (Figure 1).

The median duration of treatment with sonidegib was six months (range 3–19). Regarding the efficacy variables, it was determined that there was a total response in 9/38 patients (23.7%), partial response in 25/38 patients (65.8%) and no response in 4 patients (10.52%). None of these 4 cases had previously received Vismodegib treatment, and all had an aggressive histotype.

The mean objective response time was 6.43 months (range 2–19 months). The mean time to onset of response (considering only those patients with total or partial response) was 3 months (range 1–11 months).

In 6/34 patients, the dose was reduced to 200 mg every other day until it was discontinued due to adverse effects. The main adverse effects reported were dysgeusia (*n* = 8), asthenia (*n* = 6), muscle spasms (*n* = 6), alopecia (*n* = 4) and gastrointestinal intolerance (*n* = 4).

Finally, it was determined that there was control of the disease in 86.85% of the patients and progression in 13.15% of the cases evaluated.

## 4. Discussion

This study presents the data from our experience in real clinical practice with the use of Sonidegib in the management of locally advanced basal cell carcinoma (BCC) in an elderly population (mean age greater than 75 y.o.), with two cases in patients with Gorlin syndrome and good results of partial and total response rates with data consistent with the pivotal clinical trial (BOLT) [11] and the clinical practice series actually published.

Sonidegib was approved by the Food and Drug Administration (FDA) and by the European Commission after evaluation by the European Medicine Agency (EMA), in July and August 2015, respectively. It is indicated for the treatment of adult patients with CBCla who are not amenable to curative surgery or radiotherapy.

The efficacy and safety of sonidegib in basal cell carcinoma have been evaluated in a phase I study (X2101), in a pivotal phase II study (A2201) and in two clinical studies with a total of 62 pediatric patients (CLDE225X2104 and CLDE225C2301) [11].

A total of 230 patients were included, 194 (84.3%) with locally advanced basal cell carcinoma (LA-BCC), 36 (15.7%) with metastatic basal cell carcinoma (mBCC) and 16 presented a diagnosis of Gorlin syndrome. They were randomized to receive sonidegib at the 200 mg QD or 800 mg QD dose until disease progression or unacceptable toxicity occurred. Dose adjustments were allowed; in the 800 mg arm, a maximum of two dose reductions were allowed, and, for the 200 mg arm, only one dose reduction was allowed (placebo was administered). If a second dose reduction in this arm were necessary, treatment should be discontinued.

The main inclusion criteria were as follows: patients ≥18 years of age with a confirmed diagnosis of LA-CBC or mCBC and measurable disease; who were not candidates for radiotherapy, surgery or other local treatments; with ECOG performance status ≤2; with good renal, hepatic and spinal cord function. Ineligible patients include those with surgery in the previous 4 weeks and who had previously received sonidegib or other Hh pathway inhibitors (Hi), who could not take the medication orally, those with neuromuscular disorders or concomitant treatment with drugs that may cause rhabdomyolysis or antineoplastic treatment at the same time or within 4 weeks of initiation of treatment with sonidegib and pregnant and lactating women. Women of childbearing potential had to be using effective contraception and had to have a negative pregnancy test during the study.

Patients were stratified according to disease stage (LA-BCC vs. mBCC), histologic subtype (nonaggressive vs. aggressive for patients with aCBC) and by region (Australia, Europe and North America).

The primary efficacy endpoint was the objective response rate (ORR) according to modified response evaluation criteria in solid tumors (mRECIST) in patients with LA-BCC. Secondary endpoints were the duration of response (DR) and the complete response rate (CRR), both assessed by a central reviewer.

Baseline patient characteristics were similar between study arms and comparable to the general BCC patient population. For the total population included, the median age was 66 years (range: 24–93), and 54.3% were 65 years or older. In our study and in comparison with the data from the pivotal clinical trials, the mean age is higher in real clinical practice, and it is possibly due to the financing conditions that have been imposed in each of the hospitals participating in this study (media: 76.23 y.o.).

Furthermore, 79.9% of the patients with LA-BCC had received previous treatments: 78.9% surgery, 7.7% radiotherapy and 4.6% antineoplastic treatments (3.6% one treatment and 0.5% two previous treatments). However, the percentage of patients who had not received any prior treatment was 28.95%, being equivalent to that reported in the BOLT study.

In the first data cut, the median follow-up time was 13.9 months. Of the 230 patients included, 39 patients (49.4%) in the sonidegib 200 mg arm continued treatment; none (0%) had died; 15 (19%) had progressed; and 16 (20.3%) patients had discontinued treatment due to adverse events (AEs). At the 18-month analysis, 11 patients (13.9%) in the sonidegib 200 mg arm were continuing treatment; one patient (1.3%) died; 27 (34.2%) had progressed; and 22 (27.8%) had interrupted treatment due to AE. In the first analysis, the ORR for the LA-BCC population was 47% (95% CI: 35–60) with sonidegib 200 mg.

Beyond the results reported in clinical trials, it is important to highlight the data from real clinical practice series, although there are some discrepancies in the reported results [10,11,12,13]. In the first place, and except for the Spanish series presented by Moeno Arrones [10] with more than 80 patients, it is rare that none of the series presents a high number of patients because LA-BCC usually affects older patients, residents in rural areas with difficult access to medical services or patients with a low sociocultural level who rarely consult a general practitioner or dermatologist if the pathology does not produce symptoms or functional disability. The frequency of AE in all reported series is greater than 80% of patients including ours where almost 9 out of 10 patients report some adverse effect related to the intake of Sonidegib. In fact, in all the cases reported in our series, discontinuation of treatment, as in the Herms series [13], is caused by the associated adverse effects. The adaptation of the dosage (dose reduction) to obtain better results are data not measured in all the series of real clinical practice, although our results are in agreement with what was reported by Moreno-Arrones [10]. In Table 2, we summarize the main characteristics of the different studies.

Considering safety, however, there are significant differences in reported adverse effects, although this is possibly related to the data collection method used. In the BOLT study, 94.9% of the patients who received sonidegib 200 mg had an AE, although these were mainly grade 1 or 2. In 30.4% they were grade 3/4 AE and in 28.8% grade 3 AE/4 related to sonidegib. In 13.9%, they were serious AEs, and, in 3.8%, they were serious AEs related to treatment, with rhabdomyolysis being the most frequent AEs. Moreover, 21.5% of patients on sonidegib 200 mg discontinued treatment due to AE; 36.1% required dose interruption or reduction due to AA.

Common AEs that occurred in ≥10% of patients treated with sonidegib 200 mg were muscle spasms (49.4%, grade ≥ 3: 2.5%), alopecia (43%, grade ≥ 3: 1.3%), nausea (32.9%, grade ≥ 3: 1.3%), dysgeusia (38%, grade ≥ 3: 0%), fatigue (29.13%, grade ≥ 3: 0%). Although varying the frequency, the percentage and expression of the main side effects coincide in all the studies. In our studies, in particular, no analytical abnormalities were reported.

The frequency and importance of AEs associated with the inhibition of the Hh pathway may be a limiting factor in the continuity of treatment. Dose adjustment or temporary interruption of treatment are advisable to increase patient compliance. During the BOLT trial [11], it will be verified that temporary interruptions or dose reductions did not have a negative impact on the efficacy of sonidegib. So, although patients currently starting treatment with Hh pathway inhibitors receive important health education, which can minimize the expression of some side effects, their collection in real practice clinical series is not usually as exhaustive as in the case of clinical trials.

Likewise, dose reduction strategies or episodic treatment proposals can also reduce their communication [14]. As Moreno Arrones [10] points out, it should be noted that adverse effects tend to reappear after drug reintroduction and are dose-dependent.

A fundamental question is whether there are differences between the two iHhs, vismodegib and sonidegib. There are no studies directly comparing the safety and efficacy of iHhs, and, although there has not been a comparison between trials, an unadjusted indirect comparison of efficacy between sonidegib and vismodegib was provided [15] without major differences between the two drugs. In a systematic review and meta-analysis [16] that included 22 articles to evaluate the efficacy and safety of iHh in BCC, the pooled ORR for all patients was 64.9%, implicating there is at least a partial response (z = 7.60, *p* < 0.0001) in most patients receiving SSHis. The ORR for vismodegib was 68.5% and 50.1% for sonidegib. Finally, and to conclude, we want to highlight that our study has important limitations derived from the retrospective data collection, but it does provide data that are consistent with previously published studies in terms of effectiveness and safety. We have observed important differences in the accessibility of the drug in the different participating centers in the region, which is possibly related to the interpretation of the Spanish Position Report in each hospital independently. However, it provides important evidence that allows us to deepen our knowledge and positioning of the drug, and it is necessary to explore its role in real clinical practice with the other hedgehog pathway inhibitor financed, vismodegib, and its role not only for the treatment of LA-BCC that is unresectable or that cannot be submitted to radiotherapy but also in what may be a first step before a possibility of surgical rescue. Likewise, and although it has not been possible to explore it due to the heterogeneity in data collection, it would be interesting to establish protocols to determine what type of radiological test and with what cadence it is necessary to request in the monitoring of these patients.

The benefit of continuing treatment should be periodically evaluated since the optimal duration varies for each patient. Close follow-up is required to assess recurrence, resistance to treatment and adverse effects.

## Figures and Tables

**Table 1:** Epidemiological characteristics of the patients in our series.

*n*	38 Patients (38 LA-Basal Cell Carcinoma)
**Age (years)**	76.23 (rank: 40–101)
**Sex**	Female 22 (57.90%); Male 16 (42.10%)
**Gorlin’s syndrome**	2 (5.2%)
**Dependence** for the basic activities of daily life	26.10%
**Personal history**	39.47% (Hypertension); 18.42% (Diabetes)
**Predominant histologic subtype**	92.10% (*n* = 35) Aggresive
**Previous therapy**	Surgery (31.57%; *n* = 25), Radiotherapy (15.78%; *n* = 6), Vismodegib (31.57%; *n* = 12).

In our series, 39.47% (*n* = 15) of the included patients had hypertension in their personal history, and 18.42% (*n* = 7) were diabetics. Two patients were transplanted (hepatic and renal). There were no other patients under immunosuppressive treatment.

**Table 2:** Comparative studies of treatment with Sonidegib 200 mg in real clinical practice.

	Moreno-Arrones et al. [10]	Villani et al. [12]	Herms et al. [13]	This Study
Type of study	82	54	21	38
ORR: Objective Response Rate	Real life observational study	Real life observational study	Real life observational study	Real life observational study
AE frequency	81.7%	92.6%	80.95%	89.5%
Discontinuation due to AE	68.3%	85.1%	100%	100%
Type of study	20.7%	Not assessable	10% dose reduction and 19% temporary interruption	17.64% dose reduction

## Data Availability

Data available on request from the authors.
